# Attempts to limit sporulation in the probiotic strain *Bacillus subtilis* BG01-4^TM^ through mutation accumulation and selection

**DOI:** 10.1099/acmi.0.000419

**Published:** 2023-05-26

**Authors:** Luke M. Bosnar, Anya E. Shindler, Jennifer Wood, Craig Patch, Ashley E. Franks

**Affiliations:** ^1^​ Department of Physiology, Anatomy and Microbiology, School of Life Sciences, La Trobe University, Melbourne, Victoria 3086, Australia; ^2^​ School of Allied Health, Human Services, and Sport, La Trobe University, Melbourne, Victoria 3086, Australia; ^3^​ Vernx Pty Ltd, Level 17, 40 City Road, Southbank, Victoria 3066, Australia

**Keywords:** necrotizing enterocolitis, sporulation, *Bacillus subtilis*, mutation accumulation, serial batch culture, mutation selection

## Abstract

The use of bacterial spores in probiotics over viable loads of bacteria has many advantages, including the durability of spores, which allows spore-based probiotics to effectively traverse the various biochemical barriers present in the gastrointestinal tract. However, the majority of spore-based probiotics developed currently aim to treat adults, and there is a litany of differences between the adult and infant intestinal systems, including the immaturity and low microbial species diversity observed within the intestines of infants. These differences are only further exacerbated in premature infants with necrotizing enterocolitis (NEC) and indicates that what may be appropriate for an adult or even a healthy full-term infant may not be suited for an unhealthy premature infant. Complications from using spore-based probiotics for premature infants with NEC may involve the spores remaining dormant and adhering to the intestinal epithelia, the out-competing of commensal bacteria by spores, and most importantly the innate antibiotic resistance of spores. Also, the ability of *Bacillus subtilis* to produce spores under duress may result in less *B. subtilis* perishing within the intestines and releasing membrane branched-chain fatty acids. The isolate *B. subtilis* BG01-4^TM^ is a proprietary strain developed by Vernx Biotechnology through accumulating mutations within the BG01-4^TM^ genome in a serial batch culture. Strain BG01-4^TM^ was provided as a non-spore-forming *B. subtilis *, but a positive sporulation status for BG01-4^TM^ was confirmed through *in vitro* testing and suggested that selection for the sporulation defective genes could occur within an environment that would select against sporulation. The durability of key sporulation genes was ratified in this study, as the ability of BG01-4^TM^ to produce spores was not eliminated by the attempts to select against sporulation genes in BG01-4^TM^ by the epigenetic factors of high glucose and low pH. However, a variation in the genes in isolate BG01-4-8 involved in the regulation of sporulation is believed to have occurred during the mutation selection from the parent strain BG01-4^TM^. An alteration in selected sporulation regulation genes is expected to have occurred from BG01-4^TM^ to BG01-4-8, with BG01-4-8 producing spores within 24 h, ~48 h quicker than BG01-4^TM^.

## Introduction

The development of the intestinal disease necrotizing enterocolitis (NEC) is characterized by excessive inflammation within the intestines and has a pronounced rate of incidence in premature (<36 weeks) infants due to low intestinal microbial diversity [1–3]. During the first weeks of life, the intestinal microbiota of a full-term infant is reminiscent of the maternal oral, placental and amniotic microbiomes as a result of the successful mother–infant microbial transfer *in utero* [4–6]. The mother–infant microbial conduit ensures that the full-term infant’s intestinal microbiome is colonized with a relative level of commensal bacteria, such as *Bacteroides* spp., *Bifidobacterium* spp. and *Propionibacterium* spp.*,* which are seen to indirectly mediate intestinal health and allow the infant to develop a stable intestinal microbiome post-natally through the production of short-chain fatty acids (SCFAs) [7, 8]. Enumeration of the foetus intestines with branched chain fatty acids (BCFAs) occurs *in utero* via the foetus’ ingestion of vernix caseosa, and is a pivotal process involved in the mother–infant transfer of microbial communities due to many of the bacteria passed on from the mother to the infant requiring and utilizing BCFAs [1, 9–11]. As a result of a premature birth (<36 weeks), an infant does not ingest vernix caseosa *in utero* and consequently does not have the appropriate levels of BCFAs within the intestines to support the colonization of certain BCFA commensal bacteria [1, 12, 13]. As low levels of BCFAs are consumed by a premature infant (<36 weeks) *in utero*, the commensal BCFA bacteria transferred from the mother to the infant are unable to colonize a premature infant’s intestines, directly resulting in a significantly lower microbial diversity in the intestines of premature infants compared with full-term (40 weeks) infants [13–15]. Although a low species diversity is consistently seen in intestinal microbiomes of premature infants, longitudinal studies have seen increased incidences of NEC in premature infants whose intestinal microbiomes are dominated by *Proteobacteria* and *Firmicutes* and contained low levels of *Actinobacteria* [16, 17]. On the other hand, premature infants who did not develop NEC were observed with significantly higher levels of *Actinobacteria *, in particular *Bifidobacterium* spp. and *Propionibacterium* spp., within the intestinal microbiome than the preterm infants who did develop NEC [16, 18, 19]. The pathogenesis of NEC has been linked to the increased numbers of the opportunistic pathogens *Clostridium* spp. or *Klebsiella* spp., within the intestines of infants, as they are consistently recorded at elevated levels in the faeces of premature infants preceding NEC diagnosis [20–22]. Species of *Clostridium *, *C. perfringens *, *C. difficile* and *C. butyricum *, and *Klebsiella *, *K. oxytoca* and *K. pneumoniae *, are often isolated from the faecal samples of NEC-affected premature infants and have all been implicated in the pathogenesis of NEC due to enteral toxin production [23–26]. Opportunistic infections by *Clostridium* spp. or *Klebsiella* spp. occur in premature infants as the intestines of premature infants are not enriched with BCFAs *in utero* and are consequently not colonized with appropriate levels of BCFA bacteria, compared with full-term infants [25, 27]. The inability of various BCFA bacteria to colonize the premature infant intestines allows for the increased growth of the opportunistic pathogens *Clostridium* spp. and *Klebsiella* spp. As the lack of BCFA consumption by the foetus’ *in utero* results in the inability of premature infants to be effectively colonized by commensal BCFA bacteria post-natally, the use of prebiotic BCFAs or a probiotic with high levels of BCFAs could stimulate the colonization of commensal BCFA bacteria and potentially limit the pathogenesis of NEC [1, 12, 13].

As a result of the inadequate microbial colonization observed in infants with NEC, the form of probiotic must differ from the probiotics used to treat more stable and mature intestinal systems [28–31]. The microbial diversity present within the intestines of premature infants, in particular premature infants with NEC, has been observed to be considerably lower and more unstable than the intestinal microbiomes of healthy full-term infants [32–34]. There is also a large disparity between the number of full-term and pre-term infants who receive antimicrobial treatment post-natally, with the majority of premature infants undergoing antibiotic regimes compared to <10 % of full-term infants [32, 35]. The administration of antibiotics to premature infants has been linked to an increased rate of incidence of NEC, as the antibiotics intended to restrict the overgrowth of opportunistic pathogens within the intestines also kill susceptible commensal bacteria [36–40]. Antibiotics have been found to be unsuccessful in decreasing the levels of antibiotic-resistant bacteria implicated in the pathogenesis of NEC, such as *Klebsiella* spp., and spore-forming bacteria, such as *Clostridium difficile *, which produce antibiotic-resistant spores [41–43]. Despite evidence which indicates the association of NEC progression and antibiotic exposure post-natally, antibiotic regiments are still prescribed in conjunction with probiotic supplements when treating infants with NEC [40, 44–46]. The low species diversity seen in the intestines of premature infants with NEC, coupled with the antibiotic exposure post-natally, suggests that the use of a probiotic bacterium capable of developing spores for infants with NEC may result in an overgrowth of the spore-forming probiotic [32–34, 43, 45–48]. In the case of *Bacillus subtilis *, a common bacterial strain used in probiotics, various complications may arise from the introduction of *B. subtilis* spores into an NEC intestinal environment, including the spores remaining dormant and adhering to the intestinal epithelia, restricting the interaction between the intestinal epithelia and commensal bacteria [49–54]. From the high levels of BCFAs, *B. subtilis* is an ideal probiotic for the treatment of NEC in premature infants, but due to the resistance and ability of *B. subtilis* spores to adhere to the intestinal epithelia, it is expected that *B. subtilis* spores could survive antibiotic treatments and may remain dormant attached to the intestinal epithelium [52–56]. The issue of *B. subtilis* spores persisting in the intestines of infants with NEC is that *B. subtilis* may outcompete various commensal bacteria attempting to colonize the infant’s intestinal epithelium [48, 52–54]. Furthermore, the ability of *B. subtilis* to form spores under duress may not result in as much of an increase in intestinal BCFAs, as opposed to a viable load of *B. subtilis* that is incapable of producing spores [55–59]. The decreased release of BCFAs may be seen as *B. subtilis* vegetative cells initiate sporulation in response to stress, instead of perishing and releasing BCFAs into the intestinal environment after being lysed [58–61]. Although the durability and resistance qualities of bacterial spores has made them ideal candidates for probiotics, the idea that a *B. subtilis* isolate incapable of producing spores will perish within the stomach suggests that the subsequent lysing of *B. subtilis* will release BCFAs and increase intestinal BCFAs more than *B. subtilis* spores which would survive degradation [60–62].

The development of a mutant bacterium for commercial use in probiotics is subject to public scrutiny in regard to the manner in which the genetics of the bacterium are modified [63, 64]. Organisms with modified genomes are categorized by the protocol that was used to create the genetic mutation, being classified as either a genetically modified organism (GMO) or a non-genetically modified organism (non-GMO) [63, 64]. A GMO is created through the direct mutation of the genome via recombinant nucleic acid or genome editing techniques, whilst a non-GMO requires that the alteration to the genome has occurred naturally [65]. From the ability to manipulate the genome with regard to a specific biological process, the creation of GMOs is far more accurate than non-GMOs, but the use of GMOs commercially in probiotics raises issues due to the lack of public trust and understanding of such protocols used to develop GMOs [63–65]. As a result, the creation of genetically modified probiotics is seen to occur via non-GMO strategies, which involves the use of natural reproduction in combination with environmental factors to create genetically variant progeny [65–68].

In order to develop a non-GMO non-spore-forming *B. subtilis* isolate, a variation in genetic material pertaining to key sporulation processes must occur naturally [65–68]. The loss and gain of genetic material is common amongst all organisms from generation to generation but occurs at a much faster rate in bacteria due to increased generational times [69–71]. The fast generational times of bacteria allow for the acquirement of mutations by bacterial isolates in real time through the use of specific environmental factors that affect the transcription of genes [72–75]. Mutation accumulation experiments have the capacity to develop genetically variant bacteria, as mutations are acquired by bacterial isolates within the population during the protocol within the population [76–78]. Mutations in bacterial genomes can alter the structure of genomes and in some cases affect the overall expression of genes and consequently the isolate’s phenotype [79]. Variations in bacterial genomes can occur at the nucleotide level through the substitution, deletion or addition of nucleotides in the DNA, and can be neutral mutations, which confer no additional fitness in regard to the isolate’s survival, or mutations may occur within a gene that may impact the phenotype in a negative or positive manner [79]. However, alterations to the genome at the chromosomal level are seen to have far more adverse effects on the genetic and phenotypic expression of a bacterium than point mutations, as seen in chromosomal rearrangements, such as duplication and deletion events of certain coding regions [80, 81]. These chromosomal altering events can have a greater impact upon the genetic expression of a bacterial genome, as the amplification or removal of a coding region will directly result in the up- or downregulation of the gene products transcribed from those coding sequences [80, 81]. This rearrangement of chromosomes was previously believed to have occurred randomly by genetic drift, but recent work has indicated that positive selection from an environment can alter the order of genes and affect what genes are transcribed [82].

Although genetic transformation via mutation may underpin the process of natural selection, mutations that alter the phenotype do not always lead to beneficial traits [83]. Protocols which aim to modify specific characteristics of a bacterium, in particular for commercial probiotic use, may often contradict the natural action of selection, as knocking-out genes pertaining to key cellular processes, such as sporulation, antimicrobial resistance and toxin production, are of great interest to developers of novel probiotic strains. The past use of mutation accumulation techniques has been proven successful in creating genetic variation in *B. subtilis* isolates, resulting in the alteration of metabolic pathways [76, 84, 85]. The neutral accumulation of mutations has been previously observed to result in the loss of sporulation in *B. subtilis* isolates, with four of the five *B. subtilis* populations found to have become non-spore-forming due to mutational degradation [86].

As various studies have indicated the role a lack of glucose plays in initiating sporulation, it is perceivable that *B. subtilis* cultured on media with high levels of glucose would be unable to initiate sporulation in response to nutrient exhaustion [87–91]. If cultured continuously over multiple generations with high levels of environmental glucose, it can be hypothesized that an inability to activate sporulation means that genes involved in sporulation may be lost over generations [87–91]. Similar results could be expected for *B. subtilis* cultured at a low pH, which is seen to negatively impact the activity of *sigH* [92, 93]. The decreased activity of *sigH* would result in lower transcription of *sigH*-dependent sporulation genes, and subsequently genes transcribed by *sigH* may in turn become null genes and may be lost over generations [72, 92–94].

## Study rationale

In this study, we sought to determine the sporulation capacity of the new probiotic strain *B. subtilis* BG01-4^TM^, a proprietary strain developed for Vernx Biotechnology. After confirming the sporulation capacity of BG01-4^TM^, the focus of this study was to further develop BG01-4^TM^ as a non-spore-forming strain of *B. subtilis *. The key factors surrounding this project include the development of a sporulation null *B. subtilis* isolate, but there were pressing constraints on the methodology and protocols used to create the new isolate. The procedures used in this experiment ensure that any genetic variation incurred by BG01-4^TM^ isolates occurred via natural processes and are indicative of epigenetic factors created by the modified media. The previous mutation accumulation protocol which produced BG01-4^TM^ was a serial batch culture utilizing a chemically defined liquid medium, modified from media previously described in a study by Leitch and Collier [95]. The intent was to create a non-spore-forming *B. subtilis* by allowing isolates to accumulate mutations that may negatively impact sporulation. Whilst the mutation accumulation protocol which produced BG01-4^TM^ was not successful in creating a non-spore-forming isolate, the genomic analysis of BG01-4^TM^ and the wild-type BG01-WT determined that BG01-4^TM^ had undergone a genetic duplication event, with the BG01-4^TM^ genome and coding sequences (CDS) increasing by more than double the size from the parent strain BG01-WT (10490086 bp/10360 CDS; 4111688 bp/4300 CDS). The increase in genetic material was confirmed not to be due to contamination as taxonomic classification via Kraken and Krona of the BG01-4^TM^ genome determined the increase in genetic material was in unclassified *Bacillus* spp. and *B. subtilis* genes, with various mutations in genes involved in sporulation observed (Figs S1 and S2; Tables S3, S4 and S10, available in the online version of this article) [96, 97].

Despite BG01-4^TM^ being observed to produce spores, the non-sporulating phenotype had been observed in single colonies of BG01-4^TM^ when streaked and grown for extended periods of time on the solid version of the chemically defined media, previously used to serially culture BG01-4^TM^ from BG01-WT. The potential to produce a non-sporulating isolate from BG01-4^TM^ was perceived by successive single-cell bottlenecks, as recent work saw the loss of genes within bacterial genomes that had recently experienced genetic duplication events, after the continuous culturing of single colonies into an environment that diminishes the requirement of the isolate to express those genes [82].

As a result, this study aimed to grow BG01-4^TM^ in an environment with extremely high levels of glucose and low pH in order to ensure the inactivity of sporulation genes and select for mutations already present within BG01-4^TM^ that may negatively impact sporulation. This work opposed common practices used to create genetically variant bacteria through the use of streak dilutions on solid medium, which was utilized to guarantee the growth of monoclonal colonies from single cells, in attempts to create a new non-sporulating BG01-4^TM^ isolate. A monoclonal colony was required for the reinoculation in this study, as a genetic variance required to impair sporulation will occur through deleterious alleles, which may be lost in a larger bacterial population. The importance of isolating samples from single colonies throughout this mutation selection protocol is to ensure that the epigenetic constraints of low pH and high glucose have acted upon a single colony, in turn allowing the introduction of a single-cell bottleneck onto the corresponding plate. A recent study has demonstrated that increased levels of genetic drift and gene loss occur within bacterial genomes that have previously experienced genetic duplication events [82]. This work suggests that due to duplication in genetic material in BG01-4^TM^ genes involved in sporulation are lost via the continuous single-cell bottlenecking on media of that would not allow sporulation to be initiated. Whilst the concepts in these experiments originated from the idea of natural selection, the aim opposes natural selection, as during this protocol we attempt to decrease the fitness of *B. subtilis* by creating a non-spore-forming *B. subtilis* isolate.

Moreover, issues may arise with maintaining both a low pH and high glucose external environment within large colonies of *B. subtilis *, as large colonies of *B. subtilis* have been observed to indirectly modify the pH and glucose levels in the external environment [98–100]. In a study by Dervaux *et al*. [98], it was demonstrated that over the course of the experiment (48 h) the pH of the media had increased from the initial reading of pH 7.5 to over pH 9 [98]. Their study indicated that the ability to modify external pH by *B. subtilis* was a result of the export of an alkaline product produced by *B. subtilis* during the formation of biofilms [98]. The Dervaux *et al*. study indicates that in a larger population pH may become more alkaline as the potential for a biofilm to be formed would be greater than in a single colony, if grown with adequate nutrients, surface area and lack of interspecies competition [101, 102]. In addition, the utilization of glucose would be far greater within large populations than in single colonies, as a larger population will need to have fermented more glucose to exhibit increased growth rates [103]. Unfortunately, the increased growth rate and higher glucose utilization would decrease environmental glucose and limit the epigenetic effects of high glucose against sporulation genes within a large population opposed to single colonies, which will also still be exposed to uncolonized media [100, 103, 104].

The creation of a non-GMO *B. subtilis* isolate that is unable to produce spores will have great potential within the probiotic market, in particular for consumers with a lower immunity or poor intestinal microbiome, where spores may become an issue within the intestinal environment. This study will also provide insight into the genetic variability that may affect sporulation in *B. subtilis *.

## Methods

### Serial batch culturing of BG01-4^TM^ from BG01-WT

BG01-4^TM^ is a proprietary strain developed for Vernx. BG01-4^TM^ was developed through serial culturing of the wild-type strain *B. subtilis* BG01-WT, isolated from the gastrointestinal system of *Apis mellifera* (honey bee). The serial batch process occurred in a Sartorius glass autoclavable bioreactor for over 692 h to produce BG01-4^TM^, utilizing a modified chemically defined medium described in a study by Leitch and Collier [95] (their personal communication).

### BG01-4^TM^ and BG01-WT DNA extraction protocol

The BG01-4^TM^ and BG01-WT isolates were streaked and grown on blood agar (Table S2) for 48 h at 37 °C. DNA was then extracted from BG01-4^TM^ and BG01-WT, and then prepared with the Zymo Research fungal/bacterial DNA miniprep kit (Supplementary Information 1). The DNA was then further prepared using a QIAseq FX DNA Library Kit [24] (Supplementary Information 2) for next generation sequencing (NGS) on an Illumina Miseq sequencing machine.

### Genome annotation protocol

The BG01-4^TM^ and BG01-WT genomes were assembled with the A5 miseq pipeline, and the assembled genome was uploaded to a genome annotation server, Prokka (via https://usegalaxy.org.au), with default parameters and the input of the genus (* Bacillus *) and species names (*subtilis*) (Galaxy Version 1.14.6) (Supplementary Information 3). The BG01-4^TM^ and BG01-WT genomes were analysed for genes involved in sporulation regulatory systems, including the sporulation delay operon (*sdp*) and the sporulation killing factor operon (*skf*) (Table S10).

### Genome contamination protocol

To establish what genetic material has contributed to the increase in the genome size of BG01-4^TM^, both isolate genomes (BG01-4^TM^ and BG01-WT) were run through the taxonomic classifier Kraken (via Galaxy Version 1.14.6), which produced independent Kraken reports for each genome (Tables S1 and S2) [96]. These Kraken reports were then run through Krona Pie Chart (via Galaxy Version 1.14.6), which allows for a representation of the taxonomic classifications of a genome via an interactive pie chart (Figs S1 and S2) [97].

### Spore production protocol

*B. subtilis* BG01-4^TM^ and the control *B. subtilis* HU58 (Table S1), a known spore producer, were plated on both 2× Schaeffer-Glucose (2× SG) agar (Table S5) and incubated at 37 °C (Protocol 1.4).

### Spore examination protocol

Both BG01-4^TM^ and HU58 were examined at 24 h periods (for 3–4 days) via the Schaeffer–Fulton microscopic staining protocol to determine if the culture was only just vegetive cells, or if the commencement of sporulation had occurred, which would be seen by the presence of endospores and/or free-spores in the sample (Protocol 1.5). Plates of both BG01-4^TM^ and HU58 were examined via the Schaeffer–Fulton microscopic staining protocol (Table S6) at 24 h timepoints until free-spores make up >90 % of the culture, or until commitment and production of spores had not occurred. A failure to induce sporulation will be noted at a timepoint of approximately 96–120 h (4–5 days) with no spores being present in either endospore or free-spore form, and the vegetative cells have begun to perish due to nutrient exhaustion and the culture is predominantly cellular debris (Figs S3–S6).

### Spore purification protocol

The spores produced by BG01-4^TM^ and the control HU58 were extracted and purified by a protocol previously described [105, 105]. Adjustments to the protocol were made and included the use of a 4 °C freezer room in lieu of a 3 °C freezer room. Heat treatment of spores may be necessary after resuspending washed spores in 0.1 % lysozyme + phosphate buffer (Table S7) to completely eradicate vegetative cells. Heat treatment was done by heating the spore suspension in a 70–75 °C water bath for 10 min (Protocol 1.6)

### Spore quantification protocol

Once the spores produced by BG01-4^TM^ and the control HU58 had been purified, the quantity of spores being produced by both isolates was determined through the use of a haemocytometer (haemocytometer loading instructions: Fig. S7; haemocytometer quantifying instructions: Fig. S8) and light microscope. Amendments were made to the protocols given in other studies due to the use of a light microscope rather than a phase-contrast microscope. In this study, under a light microscope, the spores were required to be stained to be examined (Protocol 1.7).

### Selecting for sporulation defective mutations in BG01-4^TM^ isolates

BG01-4^TM^ was grown independently 15 times on 15 differently modified types of Luria–Bertani (LB) agar with different glucose molarity (200, 400 and 600 mM) and pH levels (pH 5–9) (Table S8) for multiple generations over 14 days (~400 generations) (Table S9). Over the course of the mutation selection protocol (14 days), the amount of growth by BG01-4^TM^ was recorded prior to daily re-inoculations. The level of growth by BG01-4^TM^ over a range of glucose molarities (200, 400 and 600 mM) and pH levels (pH 5–9) was determined as the high sugar levels and pH range of pH 5–9 are reminiscent of conditions found within the infant gastrointestinal tract. The amount of growth was measured by the extent of growth across the plate within 24 h: total plate coverage 90–100 %; growth on all streaks 70–90 %; growth on 2/3 streaks 50–69 %; growth on 1/3 streaks 20–49 %; growth on inoculation site 5–19 %; no growth 0 %.

This experiment was done in triplicate for a total of 45 isolates. The 45 isolates of BG01-4^TM^ (triplicates) were grown at 37 °C on one of the 15 types of media (i.e. pH 7, 400 mM), with the isolates being plated onto the same type of plate after 18–24 h of growth for 14 days. Information about media production is given in the media section. The serial batch culture procedure was done aseptically. Prior to and after each inoculation of BG01-4^TM^ with the wired loop, the wired loop was flamed for 5–10 s with a Bunsen burner to ensure the removal of any bacterial culture from the loop. From the serial batch culture, hypothetically 45 new strains of *B. subtilis* have been produced and are accordingly labelled below (Table S9).

1. BG01-4^TM^ was inoculated onto a Day 1 LB agar plate via streak dilution, with a specific pH (pH 5–9) and glucose molarity (i.e. pH 7–400 mM), and incubated at 37 °C for 18–24 h (this was replicated three times for each isolate and media type).
2. After 18–24 h of growth BG01-4^TM^ was isolated from the plate (from a single colony – if not possible, the edges of a larger colony) and re-inoculated onto a new corresponding LB agar plate and incubated again at 37 °C for 18–24 h (i.e. Day 1 pH 7–400 mM → Day 2 pH 7–400 mM).
3. Step 2 was repeated for the 15 isolates and 15 types of media until the completion of Day 14 (Table S9).
4. After 14 days of continuous growth, the 15 isolates were removed from the plates via scraping the colonies to the edge of the plate with an aseptic wired loop and pipetting LB broth onto the scraped colonies and re-pipetting up the LB broth to remove the colonies.
5. The 15 isolates were pipetted into tubes labelled with the specific strain and replicate names and stored separately in 1400 µl of LB broth and 600 µl glycerol (30 % glycerol) to ensure cells do not perish during storage in the −80 °C freezer. Tubes were vortexed prior to freezing.

## Results and discussion

### BG01-4^TM^ is a spore former

**Fig. 1.**
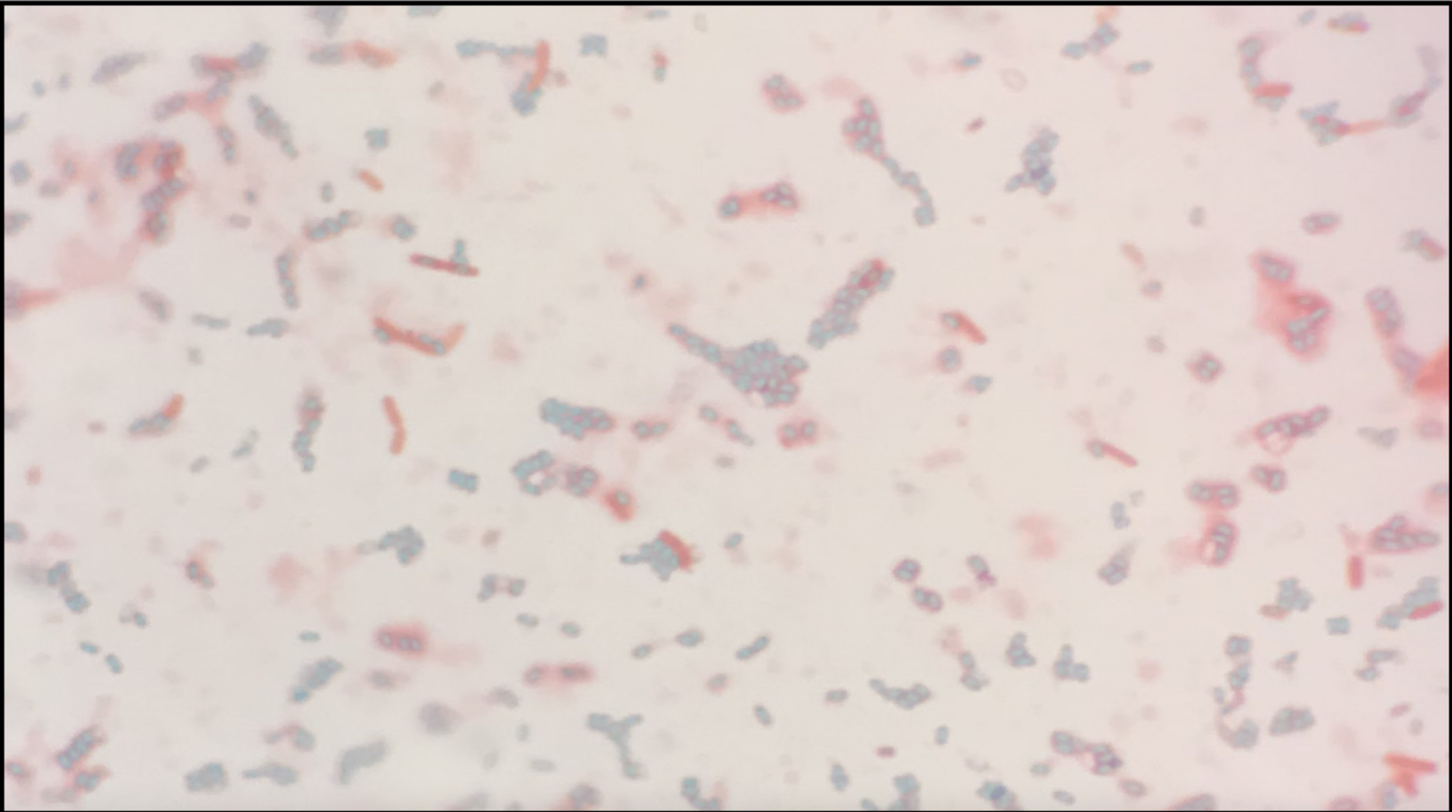
Image of spore, endospore and vegetative cell forms of *Bacillus subtilis* BG01-4^TM^. Cultured on 2× SG agar for 92 h; ×1000 magnification. Spores and endospores are stained with malachite green stain and appear light blue/green; vegetative cells are stained with safranin and appear pink/red.

BG01-4^TM^ has been established as a spore former, producing spores in response to glucose exhaustion after 92 h when cultured on 2× SG agar (Fig. 1). The *B. subtilis* isolate BG01-4^TM^ was expected to be capable of sporulation, as past studies have revealed as many as 500 genes are expressed by *B. subtilis* during sporulation [72].

Once BG01-4^TM^ was determined to form spores in response to nutrient exhaustion, the efficacy of BG01-4^TM^ to execute the process of sporulation was assessed by quantifying the spores produced by BG01-4^TM^ at a specific time-point, compared with a commercially available and known spore-forming probiotic strain, *B. subtilis* HU58 [73]. BG01-4^TM^ demonstrated a greater capacity to produce spores than HU58, calculated via a haemocytometer after 92 h of growth on 2× SG agar, with the BG01-4^TM^ sample observed to contain 37 000 000 spores ml^–1^ (3.7×10^7^ c.f.u. ml^−1^), and the HU58 sample containing 27 000 000 spores ml^–1^ (2.7×10^7^ c.f.u. ml^−1^) [74, 75]. These results did not indicate a major disparity in the number of spores produced by BG01-4^TM^ and HU58 over 92 h, but the number of spores produced by both BG01-4^TM^ and HU58 were seen to be considerably lower than a previously quantified laboratory strain, *B. subtilis* 1012 (6.7×10^8^ c.f.u. ml^−1^ after 72 h) [52]. However, a variation in the number of spores produced by a strain of *B. subtilis* is expected to occur from strain to strain, and also past studies have established that a fluctuation in the number of spores produced by *B. subtilis* will be seen on different types of media used to illicit sporulation (solid or liquid) and the original number of cells used to inoculate the culture [52]. Earlier *B. subtilis* spore quantification experiments also determined the method of spore purification to be a factor affecting the sum of spores quantified, but the lysozyme spore purification method used in this study has been regarded as one of the most effective with regard to spore purity and yield (Protocol 1.6) [52, 94].

### BG01-4^TM^ growth patterns in non-sporulation selective media

As a result of BG01-4^TM^ expressing the phenotypic ability to produce spores in response to nutrient exhaustion, we attempted to develop a non-GMO BG01-4^TM^ variant with the inability to develop spores. *B. subtilis* sporulation has been extensively studied throughout the literature, revealing environmental glucose and pH levels as key factors affecting sporulation in *B. subtilis* isolates [66–71]. As past studies have indicated the negative effects on sporulation of high levels of glucose and a pH<6, a medium was developed utilizing these factors as epigenetic constraints against sporulation [88–93].

**Fig. 2.**
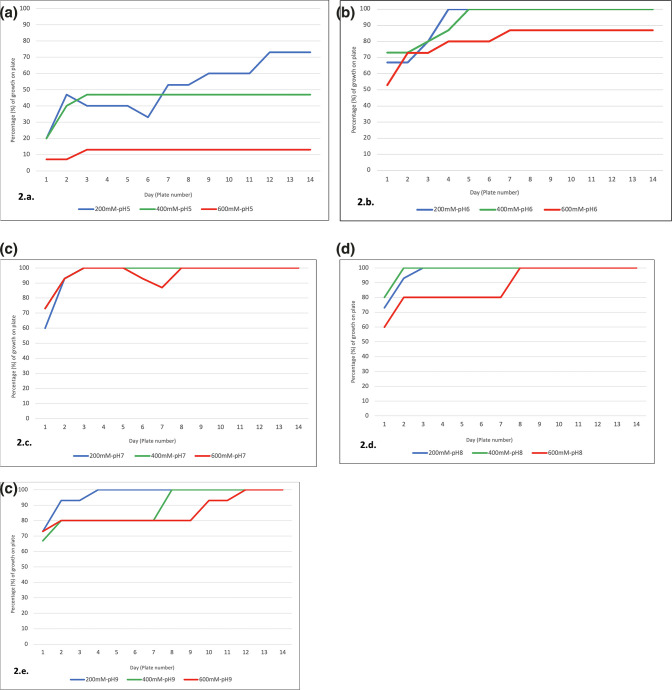
Percentage growth by *Bacillus subtilis* BG01-4^TM^ in 24 h on LB agar with excess glucose (glucose molarities: 200 mM – blue, 400 mM – green, 600 mM – red), recorded over a time period of 14 days (14 agar plates). (a) Growth at pH 5; (b) growth at pH 6; (c) growth at pH 7; (d) growth at pH 8; (e) growth at pH 9.

The mutation selection protocol of BG01-4^TM^ on LB agar with varying glucose molarities (200, 400 and 600 mM) and pH levels (pH 5–9) did not only provide the potential for a non-spore forming BG01-4^TM^ mutant to be isolated, but also the high glucose levels and relevant pH range provided the opportunity to understand where within the intestinal environment BG01-4^TM^ is most likely to thrive [106–115]. Throughout the entire selection culture (14 days) the level of growth after 24 h on the plate by the BG01-4^TM^ isolates was recorded, to determine under what glucose molarity and pH level BG01-4^TM^ exhibited the best growth (Fig. 2a–e).

BG01-4^TM^ was observed across all glucose molarities (200, 400 and 600 mM) and pH levels (pH 5–9) to have the lowest growth for each respective media type during the first 1–2 days after the initial inoculation (Fig. 2a–e). And again, across all glucose molarities and pH levels, BG01-4^TM^ demonstrated an increased growth rate for each respective media type as the day of the experiment (plate number) increased (Fig. 2a–e). BG01-4^TM^ expressed the best growth at pH 7 for all glucose molarities, establishing pH 7 as the optimum pH for BG01-4^TM^ growth (Fig. 2c). The high growth rate of BG01-4^TM^ at pH 7 is in line with other studies that have indicated that the optimum growth pH of *B. subtilis* is within the pH range 7–8 [90]. Our results further ratify a range of pH 7–8 as being the ideal pH for *B. subtilis* growth, as BG01-4^TM^ also demonstrated a high level of growth at pH 8 for all glucose molarities (Fig. 2d). Analysis of BG01-4^TM^ growth during the serial batch culture indicates that, intestinally, BG01-4^TM^ will demonstrate the highest rates of growth within the small and large intestines, which have been established to have pH ranges of pH 4–7 (Fig. 2a–e) [93, 116]. The results also suggest that at pH 7 the level of luminal glucose will not affect the rate of growth by BG01-4^TM^, which indicates that BG01-4^TM^ growth intestinally will not be greatly affected by the fluctuating glucose levels at pH 7 (Fig. 2c).

Considerable levels of growth were still exhibited by BG01-4^TM^ outside of the optimum pH range (pH 7 –8) at pH 6 and 9, with a higher growth by BG01-4^TM^ oberved at pH 6 than at pH 9 (Fig. 2b, c). Outside of the optimum pH range, an increase in glucose molarity was seen to be a factor in restricting BG01-4^TM^ growth, with 600 mM glucose at pH 6 limiting growth by ~20 %, and 400 and 600 mM glucose at pH 9 both also limiting growth by ~20 % (Fig. 2b, c). The negative effects of increasing glucose molarity on BG01-4^TM^ growth were only observed outside the optimum pH range (pH 7–8) and indicates that BG01-4^TM^ growth intestinally may be greatly impacted at pH<7 l or >8 during the following 1–2 h after feeding, as post-meal consumption will result in an increase of luminal glucose [110]. The negative impact of rising glucose molarity on BG01-4^TM^ growth outside of the pH range 7–8 suggests that if BG01-4^TM^ is consumed at around the same time as a meal, growth of BG01-4^TM^ outside of the optimum pH range (7–8) will be greatly impeded by the high luminal glucose levels [93, 116, 117]. Moreover, BG01-4^TM^ expressed the lowest growth rates at pH 5, with an increasing glucose molarity observed to further restrict growth (Fig. 2a). The growth levels of BG01-4^TM^ at pH five for all glucose molarities indicates that survival rate of BG01-4^TM^ within the stomach will be poor, and subsequently the level of viable BG01-4^TM^ vegetative cells which are able to pass through the stomach to the small intestines will be low (Fig. 2a) [102].

### Inability to knock-out sporulation in BG01-4^TM^

In order to create a non-GMO *B. subtilis* incapable of developing spores, the epigenetic factors surrounding sporulation in *B. subtilis* must be understood [118, 119]. The key genes involved in sporulation are upregulated by *B. subtilis* in response to a lack of nutrients, predominantly glucose, given that *B. subtilis* isolates demonstrated a lower rate of sporulation in environments with high levels of glucose [105, 120, 121]. Various studies have confirmed the involvement of glucose in the commitment to sporulation by *B. subtilis *, with glucose being classified as an inhibitor of sporulation and the lack of glucose an activator of sporulation in *B. subtilis* [88–91]. Transcriptional analysis of *B. subtilis* genes involved in sporulation has also revealed the negative effects of extreme pH on the expression of sporulation genes in *B. subtilis* [92, 93]. A study by Cosby *et al*. [91] revealed a lower level of sporulation by *B. subtilis* when cultured at a low pH (~pH 5) due to the decreased activity of the transcriptional factor Sigma H (*sigH*), and the subsequent decline in the transcription of *sigH*-dependent sporulation genes [92]. External glucose levels and pH have been seen to be environmental factors involved in sporulation by *B. subtilis* and have been proven to directly affect the levels of sporulation, with high environmental glucose and low pH observed to reduce sporulation in *B. subtilis* [88–93]. After 14 days of serial batch culture on the glucose and pH modified LB agar, the BG01-4^TM^ isolates (1–44, 47) (with the exception of isolates 18 and 33, which perished during the serial batch culture) were plated on 2× SG agar and it was found that all of the tested BG01-4^TM^ isolates (1–17, 19–31, 33–44, 47) had retained the ability to produce spores in response to nutrient exhaustion (Table S9).

The process of sporulation in microbes is a key survival mechanism, and in *B. subtilis* can involve the expression of over 500 genes [122]. The production of a spore by bacteria is seen to be a highly conserved system, even across different distantly divergent genera of bacteria, including *Bacillus* spp. and *Clostridium* spp. [72, 123, 124]. A list constructed by Stragier *et al*. [125] demonstrates that although variation may occur in sporulation genes, there are genes which control essential steps in the development of a spore that are highly conserved across spore-forming *Bacilli* and *Clostridia *, such as genes involved in: pre-septation, post-septation, post-engulfment and spore-coat development [73, 107, 108]. The conservation of these core sporulation genes across members of *Bacillus* spp. and *Clostridium* spp.*,* with a most recent common ancestor ~2.3 billion years ago, indicates the robustness of these genes involved in sporulation and suggests the unlikelihood of these bacteria losing these essential sporulation genes via natural processes [125]. A reason for the preservation of these fundamental sporulation genes may be in part due to the ongoing requirement of members of the genera *Bacillus* and *Clostridium* to produce spores in order to survive adverse environmental conditions [122, 125, 126]. However, the high level of conserved sporulation genes could also be attested to by the fact that during the normal vegetative growth stage the majority of sporulation genes are inactive and not being expressed, which may suggest that sporulation genes are not subject to genetic recombination and mutation during the vegetative state [125, 127]. The dormancy of sporulation genes during the normal growth stage of *B. subtilis* vegetative cells may be indicative of the inability to knock-out sporulation genes via mutation accumulation and selection protocols utilizing serial batch culture with high levels of glucose, which is designed to ensure the inactivity of sporulation genes [88, 105, 120, 121].

### BG01-4-8 regulates sporulation less than BG01-4^TM^

**Fig. 3.**
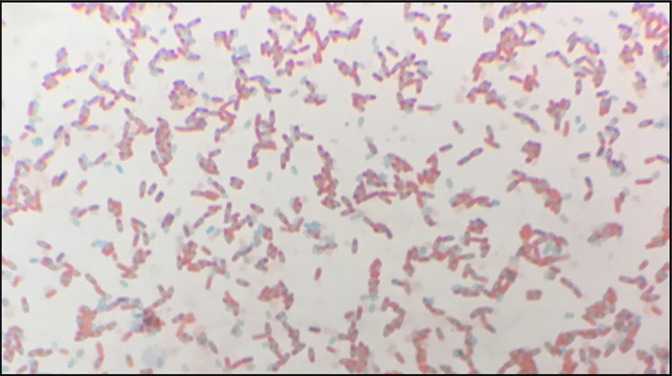
Spore, endospore and vegetative cell forms of *Bacillus subtilis* BG01-4-8. Cultured on 2× SG agar for 24 h; ×1000 magnification. Spores and endospores are stained with malachite green stain and appear light blue/green; vegetative cells are stained with safranin and appear pink/red.

Although the development of a non-sporulating BG01-4^TM^ mutant was unsuccessful, a variation in the rate of sporulation by the isolate BG01-4-8 did occur (Fig. 3). All three BG01-4-8 replicates were observed to produce spores within 24 h of inoculation on 2× SG agar (Fig. S9), ~48 h faster than the parent strain replicates, BG01-4^TM^, indicating that a potential genetic mutation has occurred regarding sporulation genes from BG01-4^TM^ to BG01-4-8 (Fig. 3) (Table S9; Figs S9 and S10).

Only BG01-4-8 and the positive control, *B. subtilis* HU58, were found to produce spores in response to nutrient exhaustion within 24 h, whilst BG01-4^TM^ and the other BG01-4^TM^ test isolates were observed to have produced spores after ~72 h (Table S9; Figs S9 and S10). The disparity in the rate of sporulation between BG01-4-8 and the other BG01-4^TM^ isolates indicates that the genetic variation which has occurred must involve genes that pertain to the regulation of the initiation of sporulation [88, 89, 128]. More precisely, as this experiment was conducted on media conducive to inducing sporulation via nutrient exhaustion, the mutation is expected to have occurred in genes involved in regulating the initiation of sporulation in response to nutrient exhaustion [88, 89, 128]. The sporulation killing factor (*skf*) and the sporulation delay operon (*sdpABC*) are expressed by *B. subtilis* to produce and export toxins (*skfA* and *sdpC*) extracellularly, which regulate community-level sporulation by targeting and killing other *B. subtilis* cells and limiting the number of cells entering sporulation [129–131]. The actions of these extracellular sporulation regulatory operons (*skf* and *sdp*) have been shown to decrease the rate of sporulation initiation when compared with *B. subtilis* mutants for the *skf* and *sdp* operons [129–132]. The *skf* and *sdp* operons slow down the initiation of sporulation by *B. subtilis* in two ways: by lysing vegetative cells with the potential to produce spores, and also by increasing the available nutrients for the vegetative cells which are not targeted, as lysed vegetative cells release cellular contents, in turn delaying nutrient exhaustion [129–131].

Genomic analysis determined that the BG01-4^TM^ genome contains the *sdp* operon, whilst being a mutant for the *skf* operon (Table S10). This indicates that BG01-4^TM^ will have a greater overall regulation of sporulation than BG01-WT, which was found to be a double mutant for *sdp* and *skf* [129–132]. A faster rate of sporulation is expected in the single mutant (*skf*) BG01-4^TM^ over the double mutant (*skf* and *sdp*) BG01-WT, as a previous study revealed faster rates of sporulation by *B. subtilis* with deletions in both the *skf* and *sdp* operons, compared with *B. subtilis* isolates with one complete *skf* or *sdp* operon [129]. As a mutation in the *sdp* operon has occurred previously in a serial batch culture protocol of BG01-4^TM^ from the parent strain, BG01-WT, it is possible that the *sdp* gene may have again been subject to a mutation during the serial batch culture of BG01-4-8 from BG01-4^TM^. If a deletion mutation has occurred in the *sdp* operon in BG01-4-8, BG01-4-8 will have become a double mutant for *skf* and *sdp*, which would be indicative of the ~48 h quicker commitment to sporulation observed by BG01-4-8 than BG01-4^TM^, which is only a single mutant for *skf* [129–132]. Although a previous study established the faster initiation of sporulation in response to nutrient exhaustion by *B. subtilis skf* and *sdp* double mutants than single mutants, to definitively determine the reason behind the ~48 h quicker initiation of sporulation by BG01-4-8 by ~48 h, the BG01-4-8 genome must be analysed and compared with the genome of BG01-4^TM^ to determine what genetic variation has occurred [129].

## Concluding remarks

A more accurate way to knock-out sporulation in BG01-4^TM^ may be required, due to the intrinsically conserved nature of core sporulation genes, even across genera of bacteria. Such methods have had pronounced results, in some cases limiting sporulation and even knocking out essential genes for sporulation in some *B. subtilis* mutants [133, 134]. Although often the development of genetic variant strains of bacteria, including *B. subtilis,* involves genome editing protocols that would classify the emerging isolate as a GMO, recent methods which utilize PCR have been proven to effectively amplify specific sections of DNA without certain genes, and are not deemed to be GMOs. Such advances and lack of public scrutiny regarding the use of these protocols suggest the potential to develop a non-spore-forming BG01-4^TM^ strain through the use of directed PCR methods [134, 135]. Due to the robustness of sporulation genes within the genome, the use of genetic engineering protocols may be the most effective way of creating a *B. subtilis* isolate that is incapable of sporulation [72, 122–124, 135, 136]. There are various genes within the *B. subtilis* genome that could be targeted to create a non-sporulating phenotype, but the genes which would most effectively negate sporulation in *B. subtilis* if removed or modified would be *spo0A*, the sporulation master regulon which has already been proven to create a sporulation null *B. subtilis* isolate when removed, and *abrB*, which is also another key transcriptional regulator in *B. subtilis,* allowing *B. subtilis* to shift its genome expression from a vegetive state to a sporulation state [137–140]. The removal or modification of either *spo0A* or *abrB* would affect the transcription of most sporulation genes, due to the fact that *spo0A* and *abrB* are seen to control the transcription of sporulation genes directly and indirectly in *B. subtilis* [137–140].

## Supplementary Data

Supplementary material 1
