# Supplementary material for: Attempts to limit sporulation in the probiotic strain Bacillus subtilis BG01-4TM through mutation accumulation and selection
Source: Access Microbiol. 2023 May 26;5(5):acmi000419. doi: 10.1099/acmi.0.000419 (PMC10267654; doi:10.1099/acmi.0.000419)

Appendix – Supplementary Information

***Bacillus subtilis* strains**

Supplementary table 1: This table displays the *Bacillus subtilis* strains used in this study, and how they were acquired.

***Bacillus subtilis* strain**	**Acquired From**
Bacillus subtilis BG01-4™	Produced by Vernx Pty Ltd; Isolated from gastrointestinal tract of *Apis mellifera*
Bacillus subtilis HU58	Microbiome Labs *Bacillus subtilis* HU58 probiotic capsule (Link under *Bacillus subtilis* HU58 access)

***Bacillus subtills* HU58 strain access**

Microbiome Labs *Bacillus subtilis* HU58 probiotic capsule purchase link:

https://shop.5ew.com.au/product/hu58/?gclid=EAIaIQobChMIzM3IrKvM9AIVgUcrCh3Siwk_EAQYASABEgI90vD_BwE

1.1. BG01-4™ and BG01-WT DNA Extraction Protocol:

Supplementary Table 2: Recipe for Nutrient Blood agar used to culture *Bacillus subtilis* BG01-4™

Media Components

Nutrient Blood Agar: For 1 L	
35 g	Blood Agar
5 g	Yeast extract
1000 ml	ddH_2O

Supplementary Information 1: Zymo Research fungal/bacterial DNA miniprep kit

Protocol

Protocol Link:

https://files.zymoresearch.com/protocols/_d6005_quick-dna_fungal

bacterial_miniprep_kit.pdf

1.2. Genome Annotation Protocol:

Supplementary Information 2: QIAseq FX DNA Library Kit (24) Protocol

Handbook and Download Link:

https://www.qiagen.com/us/products/discovery-and-translational-research/next-generation-sequencing/metagenomics/qiaseq-fx-dna-library-kit/

Supplementary Information 3: A5 Genome Assembly Pipeline

Link:

https://chipster.csc.fi/manual/a5-miseq.html

1.3. Whole Genome Contamination Protocol Supplementary Material

Supplementary Table 3: Kraken Report Taxonomic Classification of *Bacillus subtilis* BG01-WT Genome (with % of genome contributed to sequence on far left & taxonomic classification of sequence on far right).

5.05	5	5	U	0	Unclassified
94.95	94	1	-	1	Root
91.92	91	0	-	131567	cellular organisms
91.92	91	0	D	2	Bacteria
91.92	91	0	-	1783272	Terrabacteria group
91.92	91	0	P	1239	Firmicutes
91.92	91	0	C	91061	Bacilli
91.92	91	1	O	1385	Bacillales
89.90	89	0	F	186817	Bacillaceae
89.90	89	0	G	1386	Bacillus
89.90	89	0	-	653685	Bacillus subtilis group
87.88	87	6	S	1423	Bacillus subtilis
59.60	59	3	-	135461	Bacillus subtilis subsp. subtilis
40.40	40	40	-	1192196	Bacillus subtilis subsp. subtilis str. BSP1
16.16	16	16	-	1052588	Bacillus subtilis subsp. subtilis str. RO-NN-1
17.17	17	17	-	936156	Bacillus subtilis BSn5
4.04	4	0	-	96241	Bacillus subtilis subsp. spizizenii
3.03	3	3	-	655816	Bacillus subtilis subsp. spizizenii str. W23
1.01	1	1	-	1052585	Bacillus subtilis subsp. spizizenii TU-B-10
1.01	1	0	-	86029	Bacillus subtilis subsp. natto
1.01	1	1	-	645657	Bacillus subtilis subsp. natto BEST195
1.01	1	0	S	1402	Bacillus licheniformis
1.01	1	1	-	279010	Bacillus licheniformis DSM 13 = ATCC 14580
1.01	1	0	-	1938374	Bacillus amyloliquefaciens group
1.01	1	1	S	1390	Bacillus amyloliquefaciens
1.01	1	0	F	90964	Staphylococcaceae
1.01	1	0	G	1279	Staphylococcus
1.01	1	0	S	1283	Staphylococcus haemolyticus
1.01	1	1	-	279808	Staphylococcus haemolyticus JCSC1435
2.02	2	0	D	10239	Viruses
2.02	2	0	-	35237	dsDNA viruses, no RNA stage
2.02	2	0	O	28883	Caudovirales
2.02	2	0	F	10699	Siphoviridae
2.02	2	0	-	196894	unclassified Siphoviridae
2.02	2	2	S	547228	Bacillus phage PM1

Supplementary Table 4: Kraken Report Taxonomic Classification of *Bacillus subtilis* BG01-4^{TM} Genome (with % of genome contributed to sequence on far left & taxonomic classification of sequence on far right).

10.55	27	27	U	0	Unclassified
89.45	229	1	-	1	Root
88.28	226	0	-	131567	cellular organisms
88.28	226	2	D	2	Bacteria
85.55	219	0	-	1783272	Terrabacteria group
85.55	219	3	P	1239	Firmicutes
83.98	215	0	C	91061	Bacilli
83.59	214	3	O	1385	Bacillales
81.64	209	4	F	186817	Bacillaceae
79.30	203	9	G	1386	Bacillus
48.83	125	0	-	653685	Bacillus subtilis group
48.05	123	12	S	1423	Bacillus subtilis
28.91	74	6	-	135461	Bacillus subtilis subsp. subtilis
16.80	43	43	-	1192196	Bacillus subtilis subsp. subtilis str. BSP1
9.77	25	25	-	1052588	Bacillus subtilis subsp. subtilis str. RO-NN-1
10.55	27	27	-	936156	Bacillus subtilis BSn5
3.52	9	1	-	96241	Bacillus subtilis subsp. spizizenii
2.73	7	7	-	655816	Bacillus subtilis subsp. spizizenii str. W23
0.39	1	1	-	1052585	Bacillus subtilis subsp. spizizenii TU-B-10
0.39	1	0	-	86029	Bacillus subtilis subsp. natto
0.39	1	1	-	645657	Bacillus subtilis subsp. natto BEST195
0.78	2	1	-	1938374	Bacillus amyloliquefaciens group
0.39	1	1	S	1390	Bacillus amyloliquefaciens
18.36	47	47	S	666686	Bacillus sp. 1NLA3E
3.12	8	0	S	324767	Bacillus infantis
3.12	8	8	-	1367477	Bacillus infantis NRRL B-14911
2.34	6	2	-	86661	Bacillus cereus group
0.78	2	0	S	1428	Bacillus thuringiensis
0.39	1	1	-	29339	Bacillus thuringiensis serovar kurstaki
0.39	1	1	-	529122	Bacillus thuringiensis YBT-1518
0.39	1	1	S	1396	Bacillus cereus
0.39	1	0	S	580165	Bacillus cytotoxicus
0.39	1	1	-	315749	Bacillus cytotoxicus NVH 391-98
1.17	3	2	S	1404	Bacillus megaterium
0.39	1	1	-	1006007	Bacillus megaterium WSH-002
1.17	3	0	S	79885	Bacillus pseudofirmus

1.17	3	3	-	398511	Bacillus pseudofirmus OF4
0.78	2	0	S	86665	Bacillus halodurans
0.78	2	2	-	272558	Bacillus halodurans C-125
0.39	1	0	G	29331	Amphibacillus
0.39	1	0	S	1449	Amphibacillus xylanus
0.39	1	1	-	698758	Amphibacillus xylanus NBRC 15112
0.39	1	0	G	45667	Halobacillus
0.39	1	0	S	1570	Halobacillus halophilus
0.39	1	1	-	866895	Halobacillus halophilus DSM 2266
0.39	1	0	F	186818	Planococcaceae
0.39	1	0	G	648800	Solibacillus
0.39	1	0	S	76853	Solibacillus silvestris
0.39	1	1	-	1002809	Solibacillus silvestris StLB046
0.39	1	0	F	186820	Listeriaceae
0.39	1	0	G	1637	Listeria
0.39	1	0	S	1642	Listeria innocua
0.39	1	1	-	272626	Listeria innocua Clip11262
0.39	1	0	O	186826	Lactobacillales
0.39	1	0	F	81852	Enterococcaceae
0.39	1	0	G	51668	Tetragenococcus
0.39	1	0	S	51669	Tetragenococcus halophilus
0.39	1	1	-	945021	Tetragenococcus halophilus NBRC 12172
0.39	1	0	C	186801	Clostridia
0.39	1	0	O	186802	Clostridiales
0.39	1	0	F	31979	Clostridiaceae
0.39	1	0	G	1485	Clostridium
0.39	1	0	S	169679	Clostridium saccharobutylicum
0.39	1	1	-	1345695	Clostridium saccharobutylicum DSM 13864
1.56	4	0	P	1224	Proteobacteria
1.56	4	0	C	28216	Betaproteobacteria
1.56	4	0	O	80840	Burkholderiales
1.17	3	0	F	80864	Comamonadaceae
1.17	3	0	G	12916	Acidovorax
0.78	2	2	S	358220	Acidovorax sp. KKS102
0.39	1	1	S	232721	Acidovorax sp. JS42
0.39	1	0	F	119060	Burkholderiaceae
0.39	1	0	G	32008	Burkholderia
0.39	1	0	-	87882	Burkholderia cepacia complex
0.39	1	0	S	152480	Burkholderia ambifaria
0.39	1	1	-	339670	Burkholderia ambifaria AMMD
0.39	1	0	-	1783257	PVC group

0.39	1	0	P	204428	Chlamydiae
0.39	1	0	C	204429	Chlamydiia
0.39	1	0	O	1963360	Parachlamydiales
0.39	1	0	F	92713	Parachlamydiaceae
0.39	1	0	G	83551	Parachlamydia
0.39	1	0	S	83552	Parachlamydia acanthamoebae
0.39	1	1	-	765952	Parachlamydia acanthamoebae UV-7
0.78	2	0	D	10239	Viruses
0.78	2	0	-	35237	dsDNA viruses, no RNA stage
0.78	2	0	O	28883	Caudovirales
0.78	2	0	F	10699	Siphoviridae
0.78	2	0	-	196894	unclassified Siphoviridae
0.78	2	2	S	547228	Bacillus phage PM1

Supplementary Figure 1: Krona Pie Graph Displaying the Kraken Taxonomic Classifications of the *Bacillus subtilis* BG01-WT Genome

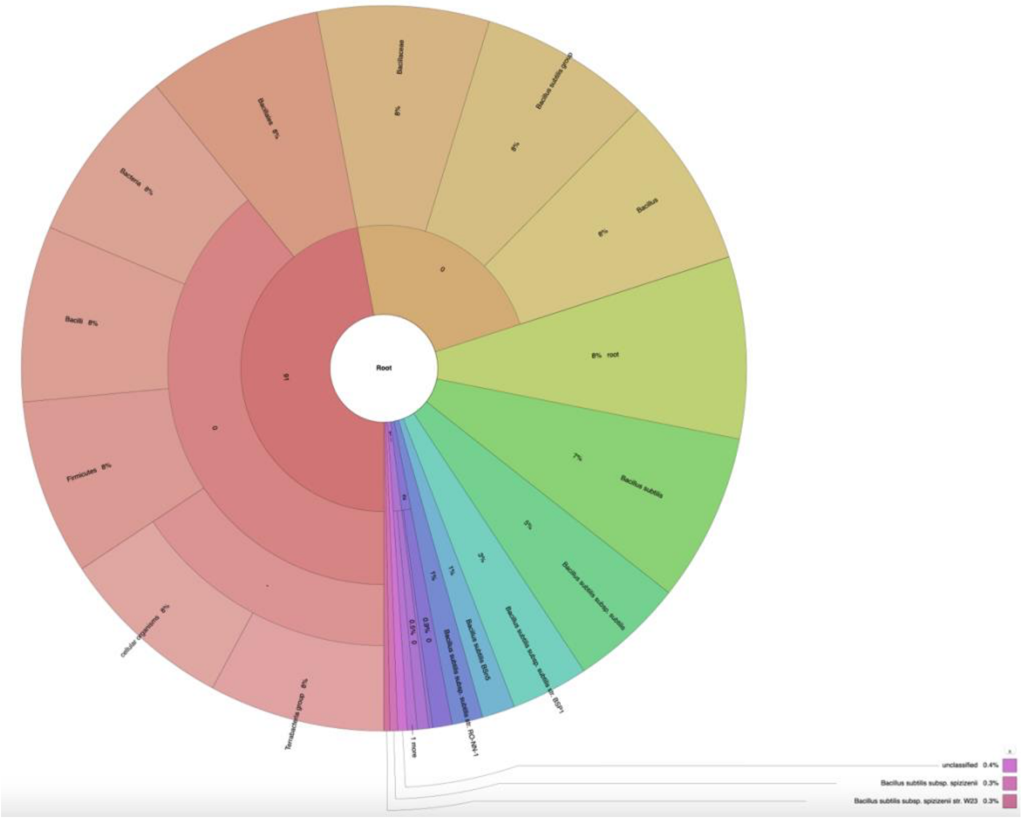

Supplementary Figure 2: Krona Pie Graph Displaying the Kraken Taxonomic Classifications of the *Bacillus subtilis* BG01-4™ Genome

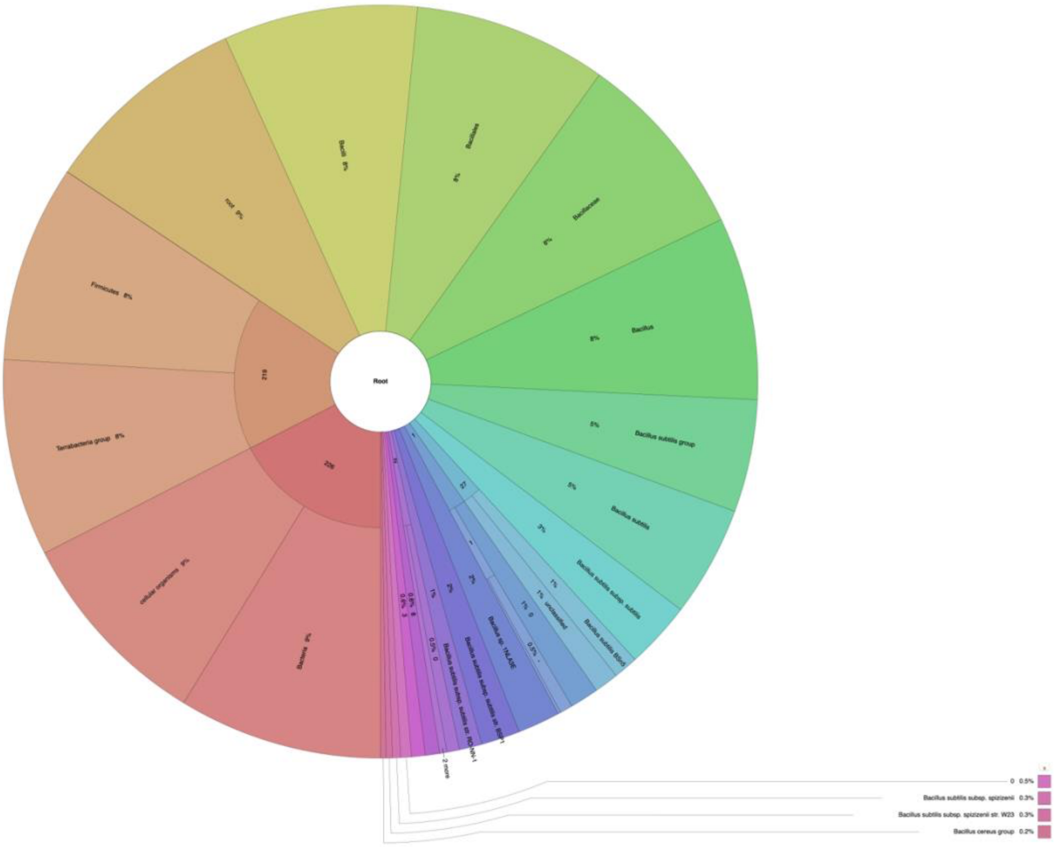

1.4. Spore Production Protocol

Supplementary table 5: This table displays the recipe for the development of 2 X Schaeffer-Glucose (2 X SG) agar, used in the spore production protocols. 2 X SG agar is a media conducive of forcing bacteria to produce spores via nutrient (glucose) exhaustion.

**2 X Schaeffer-Glucose (2 X SG) Agar Recipe (1L)**
16 g – Difco Nutrient Broth
2 g – Potassium Chloride (KCl)
0.5 g – Magnesium Sulphate ($MgSO_4$)
15 g – Agar
966.5ml – ddH_2O

1.5. Spore Examination: Schaeffer-Fulton Staining Protocol:

The *Bacillus subtilis* isolates BG01-4^{TM} and the control HU58 are examined via a differential staining method, the Schaeffer-Fulton staining protocol, which selectively stains spores and vegetative cells from a culture, which allows the determination of endospores, free-spores, and vegetative cells in a sample (See Figures 3 - 6). The Schaeffer-Fulton staining protocol follows below and has followed methodology used in other studies.

Supplementary table 6: This table displays the dyes used in this study, and what aspect of the bacterial culture (spores or vegetative cells) the dye stains.

**Bacterial Dyes**	**Stains**
Malachite Green (0.5% wt/vol)	Spores<br>(See under microscopic examination example)
Safranin Red (0.5% wt/vol)	Vegetative Cells<br>(See under microscopic examination example)

Note: The Schaeffer-Fulton staining method is conducted under aseptic procedures, with the Bunsen burner on a blue flame. Also, turn on the water bath to 60-65°C before commencing the protocol, to ensure by Step 4 that the water bath is 60-65°C. The use of the water bath is modified from other studies, where the heating of the Malachite green dye-stained slide occurred with a Bunsen burner flame.

1. BG01-4™ and HU58 are isolated from 2 X SG cultures at 24-hour time points and smeared in 5-10µl of ddH_2O on a clean glass microscope slide.

2. The smeared cultures are allowed to air dry for approximately 5 minutes, or until the smear has dried.

3. The glass slide is then heat-fixed by being passed culture side up through the flame 3 times.

4. Once heat-fixed, an absorptive piece of paper is placed over the slide and the culture is stained with the primary stain, 0.5% (wt/vol) Malachite green dye and placed upon a floating foam vessel in the water bath (60-65°C) and left to heat and stain for 5 minutes. Note: The heating of the culture is a pivotal process that softens the spore-coat and cortex, thus allowing the uptake of the stain by the spore.

5. The slide is removed from the water bath and the stained absorptive paper is removed and the slide is left to cool for 1 minute and the slide is rinsed with tap water for 30 seconds.

6. The slide is then counterstained with 0.5% safranin red dye for 1 minute.

7. The slide is rinsed for 30 seconds and gently blot dried with paper towel ensuring removal of excess water.

8. Once slides have been appropriately prepared in regard to steps 1-7, the slides are examined under a light microscope at x1000 magnification to determine the presence or lack of endospore or free spores in the culture (See Figures 3 - 6).

Microscopic Examination Examples:

<u>The interpretation of the stained slides:</u>

Spores will appear as greenish-blue spheres (See Figure 4).

Vegetative cells will appear as pink and rods (See Figures 3 - 5).

Endospores will appear as greenish-blue spheres within pink rods (vegetative cells) (See Figures 4 & 5).

Supplementary figure 3: Displays *Bacillus subtilis* BG01-4^{TM} vegetative cells stained with Safranin Red (0.5% wt/vol) dye and appear pink/red. Viewed under a light-microscope at magnification of x1000.

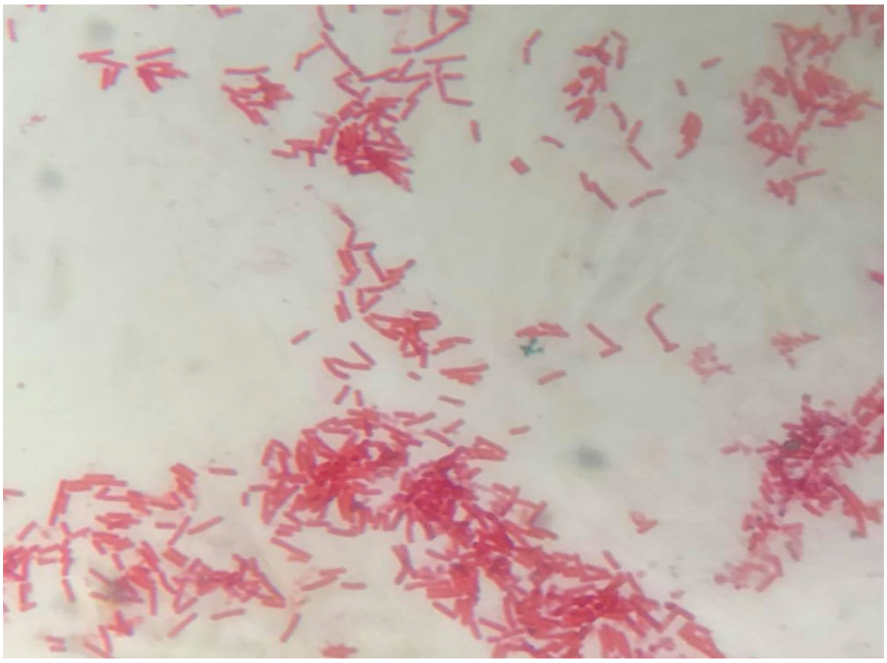

Supplementary figure 4: Displays *Bacillus subtilis* BG01-4^{TM} endospores within vegetative cells. The endospores is stained with Malachite Green (0.5% wt/vol) dye and appear light blue/green, and the vegetative cells are stained with Safranin Red (0.5% wt/vol) dye and appear pink/red. Viewed under a light-microscope at magnification of x1000.

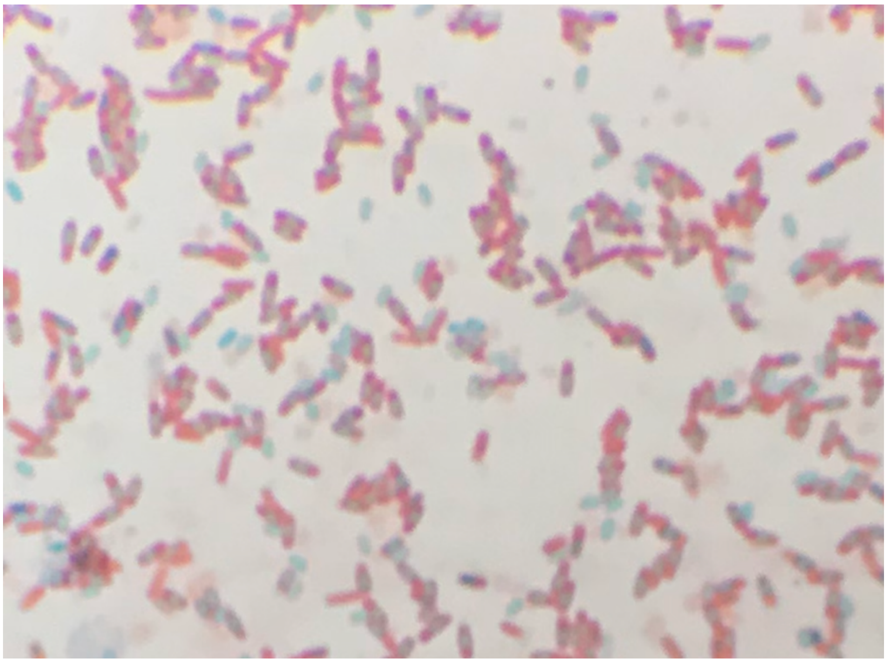

Supplementary figure 5: Displays *Bacillus subtilis* BG01-4^{TM} spores stained with Malachite Green (0.5% wt/vol) dye and appear light blue/green, and the vegetative cells are stained with Safranin Red (0.5% wt/vol) dye and appear pink/red. Viewed under a light-microscope at magnification of x1000.

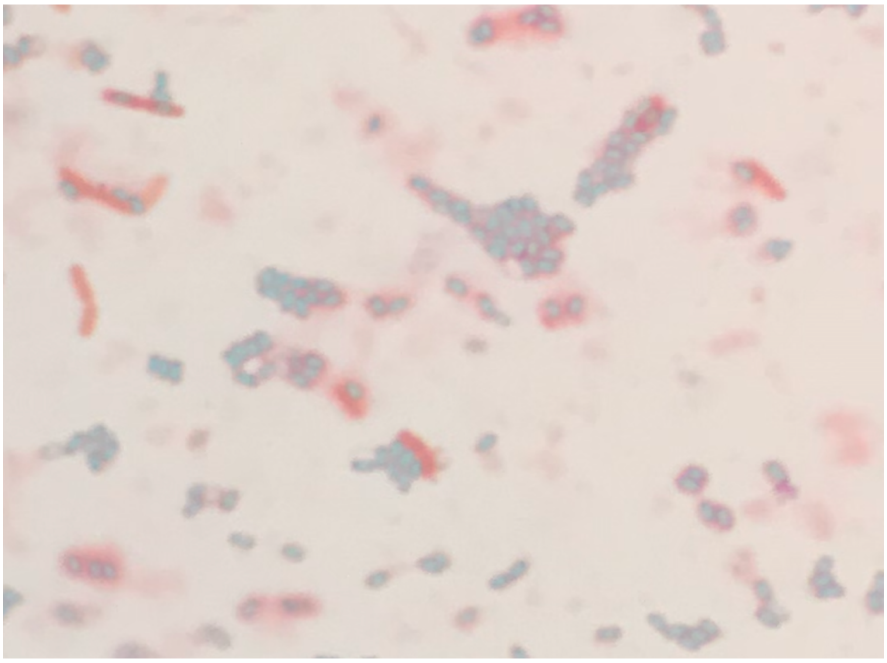

Supplementary figure 6: Displays *Bacillus. subtilis* BG01-4^{TM} cellular debris stained with Safranin Red (0.5% wt/vol) dye and appear pink/red. Viewed under a light-microscope at magnification of x1000.

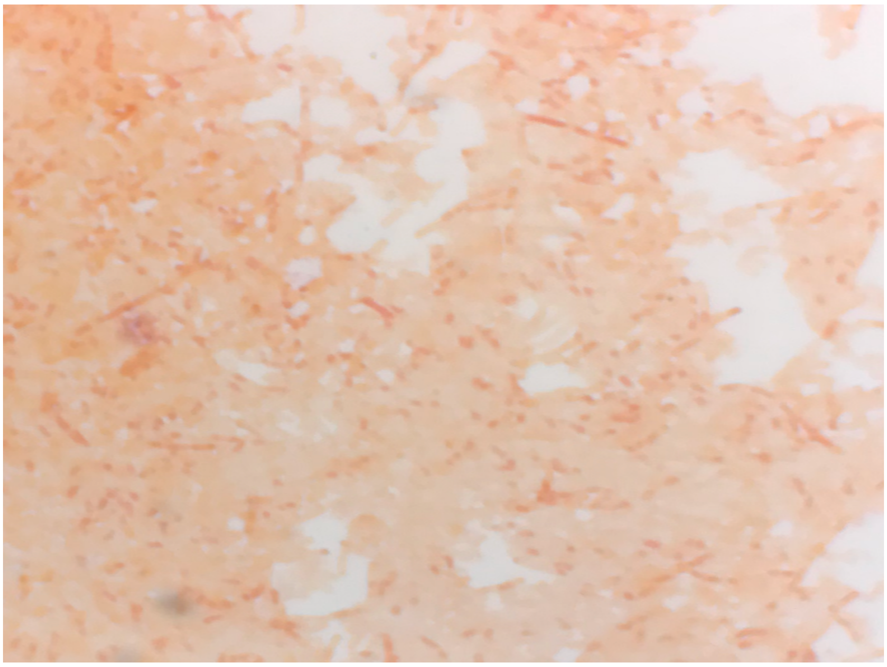

1.6. Spore Purification Protocol:

The spores produce by the *Bacillus subtilis* isolates BG01-4^{TM} and the control HU58 were extracted and purified by a protocol previously described in E.M. Powers, et. al 1968. Adjustments to the protocol were made and included the use of a 4°C freezer room in lieu of a 3°C freezer room. Heat treatment of spores may be necessary after resuspending washed spores in 0.1% lysozyme to completely eradicate vegetative cells. Heat treatment was done by heating the spore suspension in 70-75°C water bath for 10 minutes.

Supplementary Table 7: This table displays the recipe for the development of Lysozyme + Phosphate buffer, used in the spore purification protocol.

**0.1 % Lysozyme & Phosphate Buffer Recipe (100 ml)**
99.9 ml - Phosphate Buffer (PB) – 0.66 M Dipotassium Hydrogen Orthophosphate (K_2HPO_4)<br><br>(100 ml PB = 88.5 ml ddH_2O + 11.5 g K_2HPO_4)
0.1 g Human Lysozyme

1. 0.5 – 1.5 g (wet weight) of cells are suspended in 20 ml of 0.1% lysozyme, made up of filter-sterilized 0.066 M phosphate buffer (K_2HPO_4 - Dipotassium hydrogen orthophosphate) at 6.24 pH. (See Supplementary Table 7) (100ml x 0.1 = 100ml phosphate buffer + 0.1g lysozyme)
2. The cells were shaken gently at 4°C for 1 hour, until a heavy precipitate of clumped cells forms.
3. The suspension is then filtered through a sterile 10 uL polypropylene membrane filter, then the filtrate is examined microscopically.

4. Microscopic examination of stained samples should reveal the filtrate to be free of vegetative cells and a spore suspension (See Figure 9), and the material retained by filter to be mainly vegetative cells and some spores.
5. Spore suspension is washed x4 with distilled water at 7,000 rpm for 5 minutes at 4°C.
6. Washed spores were resuspended in 0.1% lysozyme.

1.7. Spore Quantification Protocol:

Once the spores produce by the *Bacillus subtilis* isolates BG01-4^{TM} and the control HU58 were purified, the quantity of spores being produced by both isolates was determined through the use of a hemocytometer and light microscope. Amendments were made to the protocols present in other studies due to the use of a light microscope and not a phase-contrast microscope. In this study, while using a light microscope, the spores were required to be stained to be examined.

Note: This is your dilution factor. The dilution factor is found out by:

Dilution Factor = Total Volume/Cell-stock volume.

Total Volume = 500 µl spore stock suspension + 10 µl Malachite green dye = 510 µl Dilution Factor = 510 (Total volume) / 500 (Cell-stock volume) = 1.02

1. 500 µl of spore stock suspension (produced in Spore Purification Protocol) with 10 µl of Malachite green dye is pipetted into a clean Eppendorf tube and vortexed for 30 seconds.
2. The spore stock + Malachite green suspension is heated in the water-bath at 60-65°C for 5-10 minutes to ensure; that the spore outer layer softens and takes up the malachite green dye, and that spore clumping is minimalised.

3. Prepare you hemocytometer before loading it by wiping down the viewing chamber with ethanol and place a clean glass coverslip over the viewing chamber.

4. Once heated and stained, approximately 10 µl of spore stock + Malachite green suspension is loaded into the hemocytometer (10 µl or until the hemocytometer viewing chamber is full).

5. After loading, wait 1 minute before beginning to count spores. This will ensure the spores within the loaded spore stock + malachite green suspension settle and stop moving, allowing easier counting of spores.

6. Once the hemocytometer has been loaded, it is placed under a microscope. Using the microscope, focus onto the grid lines of the hemocytometer at x100 magnification and identify the corner squares that are divided up into 16 smaller squares (See image).

7. With a hand tally counter (or another counting method) begin to count the spores in each of the 16 squares that make up each of the 4 corner squares (Corners: A, B, C & D). To ensure effective counting of spores in squares, employ the protocol where spores are only counted if they are within the square, or are on the right-hand and/or bottom boundary (whilst excluding spores of the left-hand and top boundary from that count).

After counting all 4 corner squares, the quantity of spores is worked out as cells per ml of solution through the formula: $((A+B+C+D)/4) \times 10^4$ x dilution factor.

Supplementary figure 7: Is an image of the haemocytometer loading area, with red arrows indicating where the cell suspension is loaded into the haemocytometer.

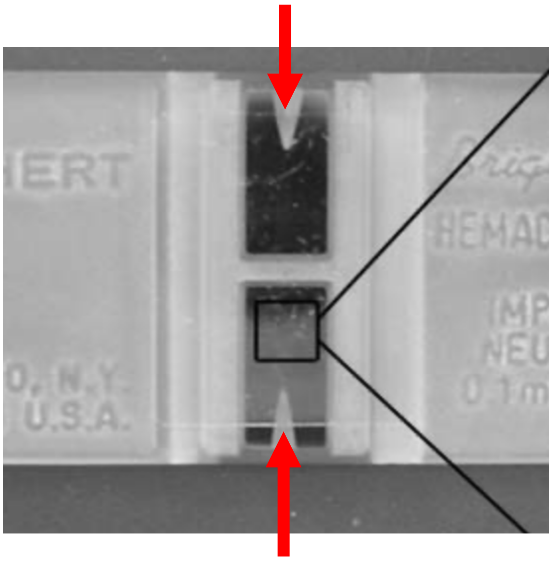

Supplementary figure 8: Displays a diagram of the counting grids (A.B. C & D) on a haemocytometer. Which are then used to calculate the colony forming units via the formula; ((A+B+C+D)/4) X 10^4 x dilution factor.

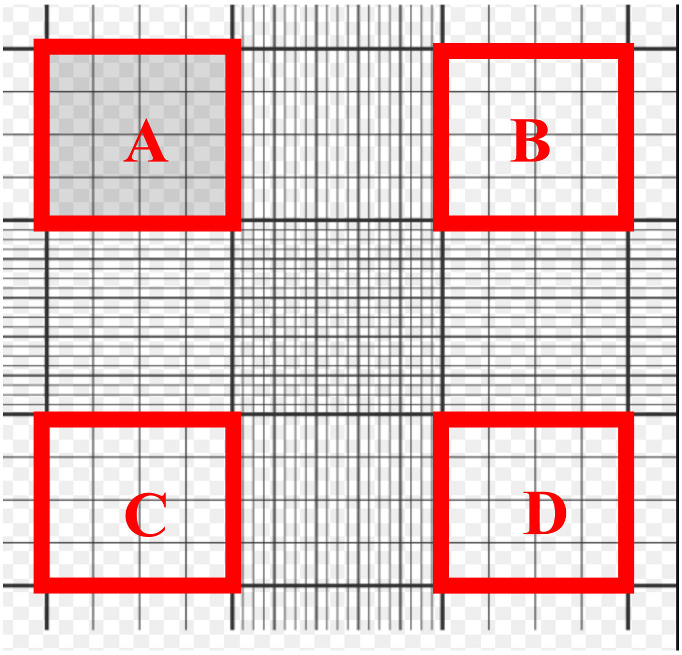

Supplementary figure 9: Displays purified BG01-4^{TM} spores (free of vegetive cells) loaded into a haemocytometer, which have been stained with Malachite Green (0.5% wt/vol) dye and appear light blue/green, under 100x Magnification.

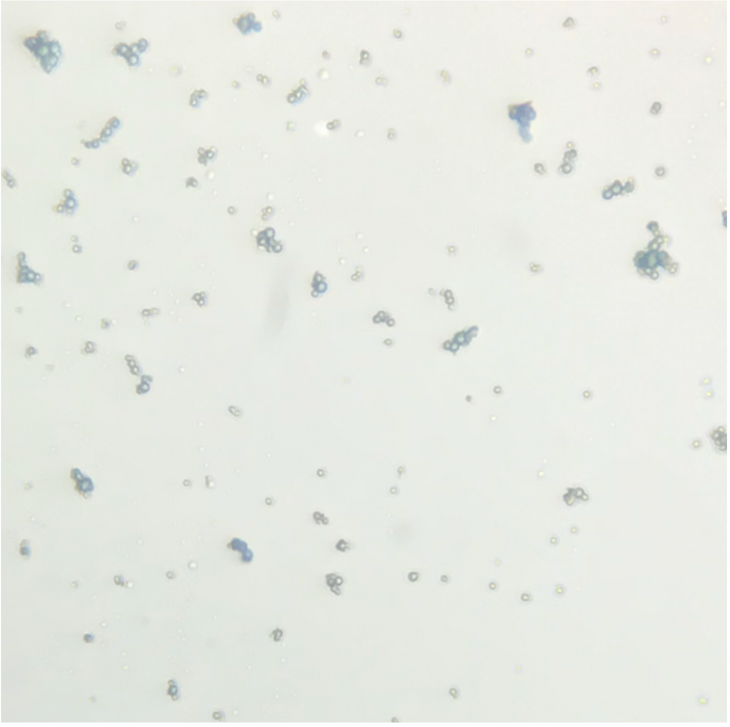

1.8. Mutation Accumulation - Serial Batch Culture Protocol:

Supplementary table 8: This table displays the recipe for the development of Luria-Bertani (LB) agar, used in the continuous culture protocol in this experiment. The relevant amounts (in grams) of glucose to be added to a 1 litre LB solution is presented for all molarities: 200mM, 400mM & 600mM, and to be added before autoclaving. The required solutions to make the appropriate increases or decreases to the pH are also listed, and to be done after autoclaving.

**Luria-Bertani (LB) Agar Recipe (1L)**	**Glucose Molarity Adjustments (1L)**	**pH Adjustments**
5 g – Yeast Extract	200mM = 36.032 g – Glucose	**To Increase pH**
10 g – Sodium Chloride (NaCl)	400mM = 72.064 g – Glucose	Add 1 M Sodium Hydroxide (NaOH)
10 g – Tryptone	600mM = 108.096 g – Glucose	**To Decrease pH**
15 g – Agar	*Done before autoclave	Add 1 M Hydrochloric Acid (HCl)
ddH_2O – amount based upon glucose mM<br>200mM – 924 ml ddH_2O<br>400mM – 888 ml ddH_2O<br>600mM - 852 ml ddH_2O		*Done after autoclave

Supplementary Table 9: Growth Conditions for Serial Batch Cultivation: Presents the type of media (growth condition), sporulation ability and rate of sporulation (hours) of the *Bacillus subtilis* isolates; the positive controls HU58 and BG01-4™, and the test isolates BG01-4 - (1- 45).

***Bacillus subtilis* strain**	**Growth Condition**	**Sporulation**	**Rate**
HU58 (control)	N/A	Yes	24 hours
BG01-4™ (control)	N/A	Yes	72 hours
BG01-4-1	200mM – pH 5	Yes	72 hours
BG01-4-2	400mM – pH 5	Yes	72 hours
BG01-4-3	600mM – pH 5	Yes	72 hours
BG01-4-4	200mM – pH 6	Yes	72 hours
BG01-4-5	400mM – pH 6	Yes	72 hours
BG01-4-6	600mM – pH 6	Yes	72 hours
BG01-4-7	200mM – pH 7	Yes	72 hours
BG01-4-8	400mM – pH 7	Yes	24 hours
BG01-4-9	600mM – pH 7	Yes	72 hours
BG01-4-10	200mM – pH 8	Yes	72 hours
BG01-4-11	400mM – pH 8	Yes	72 hours
BG01-4-12	600mM – pH 8	Yes	72 hours
BG01-4-13	200mM – pH 9	Yes	72 hours
BG01-4-14	400mM – pH 9	Yes	72 hours
BG01-4-15	600mM – pH 9	Yes	72 hours
BG01-4-16	200mM – pH 5	Yes	72 hours
BG01-4-17	400mM – pH 5	Yes	72 hours
BG01-4-18	600mM – pH 5	N/A	N/A
BG01-4-19	200mM – pH 6	Yes	72 hours
BG01-4-20	400mM – pH 6	Yes	72 hours
BG01-4-21	600mM – pH 6	Yes	72 hours
BG01-4-22	200mM – pH 7	Yes	72 hours
BG01-4-23	400mM – pH 7	Yes	72 hours

BG01-4-24	600mM – pH 7	Yes	72 hours
BG01-4-25	200mM – pH 8	Yes	72 hours
BG01-4-26	400mM – pH 8	Yes	72 hours
BG01-4-27	600mM – pH 8	Yes	72 hours
BG01-4-28	200mM – pH 9	Yes	72 hours
BG01-4-29	400mM – pH 9	Yes	72 hours
BG01-4-30	600mM – pH 9	Yes	72 hours
BG01-4-31	200mM – pH 5	Yes	72 hours
BG01-4-32	400mM – pH 5	Yes	72 hours
BG01-4-33	600mM – pH 5	N/A	N/A
BG01-4-34	200mM – pH 6	Yes	72 hours
BG01-4-35	400mM – pH 6	Yes	72 hours
BG01-4-36	600mM – pH 6	Yes	72 hours
BG01-4-37	200mM – pH 7	Yes	72 hours
BG01-4-38	400mM – pH 7	Yes	72 hours
BG01-4-39	600mM – pH 7	Yes	72 hours
BG01-4-40	200mM – pH 8	Yes	72 hours
BG01-4-41	400mM – pH 8	Yes	72 hours
BG01-4-42	600mM – pH 8	Yes	72 hours
BG01-4-43	200mM – pH 9	Yes	72 hours
BG01-4-44	400mM – pH 9	Yes	72 hours
BG01-4-45	600mM – pH 9	Yes	72 hours

Supplementary Table 10: Sporulation Regulatory Genes in the screened *Bacillus subtilis* strains BG01-4™ and BG01-WT: Displays the genes involved in the Sporulation Killing Factor (*skf*) and Sporulation Delay Protein (*sdp*) operons, and the genes from each operon that are present in each strain of *Bacillus subtilis.* Green indicates the presence of a gene and red indicates the absence of a gene.

**Sporulation Regulation:**		
**Sporulation Killing Factor**	BG01-4™	BG01-WT
skfA		
skfB		
skfC		
skfD		
skfE		
skfF		
skfG		
skfH		
**Sporulation Delay Operon**	BG01-4™	BG01-WT
sdpA		
sdpB		
sdpC		

Supplementary figure 10: Displays the triplicates (labelled 1, 2 & 3) of BG01-4-8 plated on 2 X Schaeffer-Glucose Plates after 24-hours. The white opaque colouring of the plate is an indication that the isolate is producing spores, however microscopic examination is required to confirm presence of spores (See Protocol 1.2 for microscopy protocol).

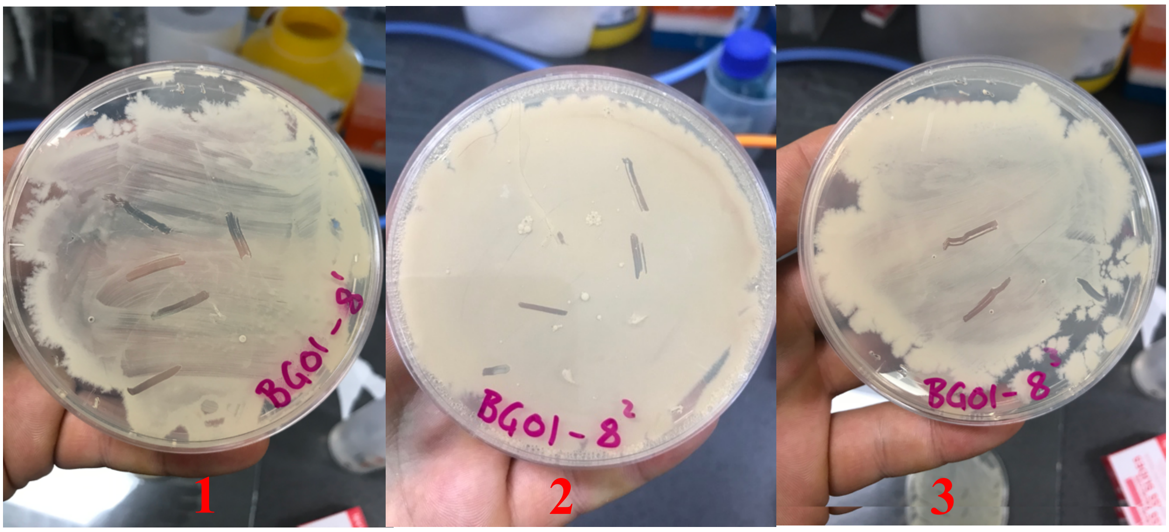

Supplement: Supplementary material 1 [file acmi-5-419-s001.pdf]
